# Capturing the biological impact of CDKN2A and MC1R genes as an early predisposing event in melanoma and non melanoma skin cancer

**DOI:** 10.18632/oncotarget.1444

**Published:** 2013-12-16

**Authors:** Joan Anton Puig-Butille, María José Escámez, Francisco Garcia-Garcia, Gemma Tell-Marti, Àngels Fabra, Lucía Martínez-Santamaría, Celia Badenas, Paula Aguilera, Marta Pevida, Joaquín Dopazo, Marcela del Río, Susana Puig

**Affiliations:** ^1^ Melanoma Unit, Hospital Clinic & IDIBAPS (Institut d’Investigacions Biomèdiques Agustí Pi i Sunyer), Barcelona, Spain; ^2^ Centro de Investigación Biomédica en Red de Enfermedades Raras (CIBERER), Barcelona, Spain; ^3^ Regenerative Medicine Unit. Epithelial Biomedicine Division. Centro de Investigaciones Energéticas, Medioambientales y Tecnológicas (CIEMAT), Madrid, Spain; ^4^ Department of Bioengineering. Universidad Carlos III (UC3M), Madrid, Spain; ^5^ Centro de Investigación Biomédica en Red de Enfermedades Raras (CIBERER), Madrid, Spain; ^6^ Functional Genomics Node, National Institute of Bioinformatics, CIPF Valencia, Spain; ^7^ Department of Bioinformatics, Centro de Investigación Príncipe Felipe, Valencia, Spain; ^8^ Biological Clues of the Invasive and Metastatic Phenotype Group. Molecular Oncology Lab, IDIBELL, Barcelona, Spain; ^9^ Tissue Engineering Unit. Centro Comunitario de Sangre y Tejidos del Principado de Asturias (CCST), Oviedo, Spain; ^10^ Centro de Investigación Biomédica en Red de Enfermedades Raras (CIBERER), Valencia, Spain

**Keywords:** Familial Melanoma, CDKN2A, MC1R, gene expression, p16, mutations

## Abstract

Germline mutations in CDKN2A and/or red hair color variants in MC1R genes are associated with an increased susceptibility to develop cutaneous melanoma or non melanoma skin cancer.

We studied the impact of the CDKN2A germinal mutation p.G101W and MC1R variants on gene expression and transcription profiles associated with skin cancer. To this end we set-up primary skin cell co-cultures from siblings of melanoma prone-families that were later analyzed using the expression array approach.

As a result, we found that 1535 transcripts were deregulated in CDKN2A mutated cells, with over-expression of immunity-related genes (HLA-DPB1, CLEC2B, IFI44, IFI44L, IFI27, IFIT1, IFIT2, SP110 and IFNK) and down-regulation of genes playing a role in the Notch signaling pathway. 3570 transcripts were deregulated in MC1R variant carriers. In particular, genes related to oxidative stress and DNA damage pathways were up-regulated as well as genes associated with neurodegenerative diseases such as Parkinson’s, Alzheimer and Huntington.

Finally, we observed that the expression signatures indentified in phenotypically normal cells carrying CDKN2A mutations or MC1R variants are maintained in skin cancer tumors (melanoma and squamous cell carcinoma). These results indicate that transcriptome deregulation represents an early event critical for skin cancer development.

## INTRODUCTION

The worldwide incidence of skin cancer, including non melanoma skin cancer (NMSC) and cutaneous melanoma (CM), is rapidly increasing, being the most common human cancer[1]. While, both entities are influenced by the carcinogenic effect of UV exposure, their incidence and tumor aggressiveness differ considerably, CM being the least frequent and most aggressive form[2].

Approximately, 10% of total melanoma cases originate in individuals belonging to high-risk melanoma pedigrees. To date, *CDKN2A* is the major gene associated with the high risk inherited in melanoma prone families [3] and in multiple primary melanoma patients [4]. *CDKN2A* acts as a tumor suppressor gene, negatively regulating cell cycle progression and promoting cellular senescence. Recently, a role for *CDKN2A* in cellular oxidative stress regulation has been suggested [5, 6]. Recurrent *CDKN2A* mutations have been identified in melanoma families [7-11], mutation p.G101W being the most frequent one detected in Mediterranean pedigrees [12]. Although heterozygous loss of *CDKN2A* is sufficient to confer a 67% lifetime risk of melanoma [13], the mechanisms responsible for tumor enhancement still have to be clarified [14]. In contrast, the role of high-penetrance *CDKN2A* mutations in NMSC susceptibility has not been clearly elucidated [15, 16].

Skin cancer epidemiology is complex due to the multigenic nature of the disease. In particular, the *MC1R* gene is a key regulator of skin pigmentation and melanocyte differentiation, playing a central role in determining the pigmentation phenotype, sun sensitivity and tanning ability[17]. Certain *MC1R* polymorphisms are responsible for the red hair color (RHC) phenotype (red hair, fair skin and poor tanning response)[18] which is associated with high UV-radiation sensitivity and skin cancer susceptibility (CM and NMSC)[19, 20]. Furthermore, *MC1R* variants are a modifying factor for melanoma risk in *CDKN2A* mutation carriers, [21-23] suggesting that multiple medium- and low-penetrance genes may influence the risk conferred by high penetrance melanoma genes.

To date, there is a lack of information about the mechanisms underlying the increased skin cancer risk in carriers of *CDKN2A* mutations in association with *MC1R* variant*s*. The effect of both genes has been separately investigated using murine-derived melanocytes and/or focused on mono-cultured melanocytes [6, 24-26]. However, these mono-cultures exhibit phenotypic characteristics closely mimicking those observed in transformed melanocytes, such as elevated growth rate [27, 28] and acquired expression of melanoma-associated antigens[29, 30] which are abolished when they are co-cultured with keratinocytes[30-32]. This data indicates that melanocyte proliferation and behavior is controlled by surrounding basal keratinocytes which regulate epidermal homeostasis [33, 34]. Thus, the establishment and characterization of a melanocyte–keratinocyte co-culture system is essential for elucidating the early molecular events leading to skin cancer.

The aim of the present study was to investigate the global molecular effect of germinal *CDKN2A* mutations (p.G101W) and *MC1R* RHC variants in the transcriptome of primary skin cells from individuals belonging to skin cancer prone families (familial melanoma pedigrees).

## RESULTS

### Impact of *CDKN2A* germinal mutation p.G101W on Global Transcript Profiles of skin primary cultures

Whole genome expression profiling was performed on melanoctye-keratinocyte co-cultures from *CDKN2A* mutation carriers (samples A1 and B1 described in Table 1) and wild-type *CDKN2A* individuals (samples A2 and B2). After stringent microarray data filtering, 1536 of the initial 19596 transcripts on the array were found to be differentially expressed in *CDKN2A* mutated cultured cells. Specifically, 60.7% of transcripts were found to be up-regulated (933/1536) and 39.3% of them down-regulated (603/1536).

**Table 1:** Genotype and phenotype features of four individuals from two melanoma pedigrees

Pedigree A						
Individual	MM*	CDKN2A	MC1R	Skin phototype	Eye color	Hair color
A1	5	p.G101W	p.R160W, p.R151C	II	Green	Red
A2	0	Wild-type	p.R160W, p.R151C	II	Green	Red
Pedigree B						
Individual	MM*	CDKN2A	MC1R	Skin phototype	Eye color	Hair color
B1	0	p.G101W	Wild-type	II	Blue	Black
B2	0	Wild-type	Wild-type	II	Blue	Brown

Two siblings from each pedigree (A and B) were included. Each individual was considered as a “genomic condition” in the study. A total of two and three melanoma cases have been found in pedigree A and B, respectively. Abbreviations: *CDKN2A*: cyclin-dependent kinase inhibitor 2A; *MC1R*: melanocortin 1 receptor; MM: melanomas.

* Number of melanomas in each individual.

**Figure 1:**
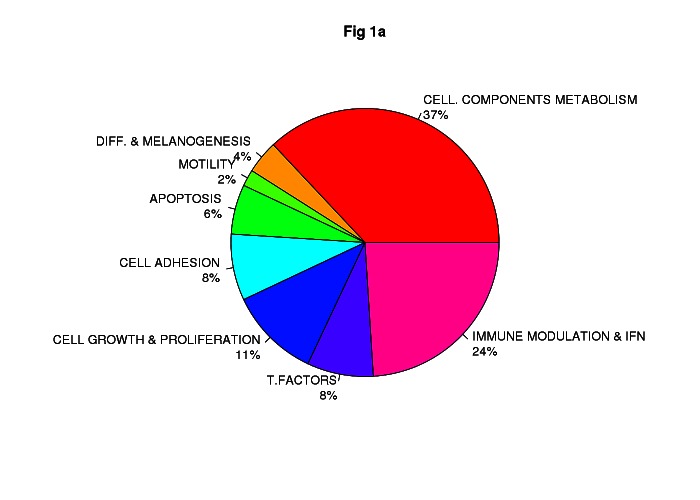

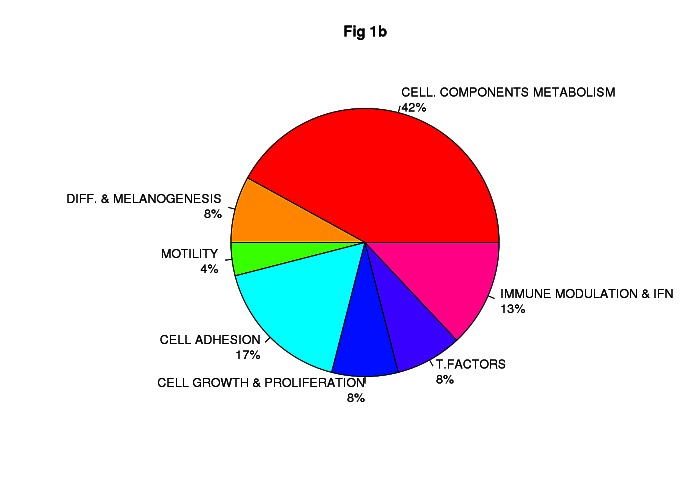
Impact of germinal CDKN2A mutation on global transcript profile in melanocyte-keratinocyte co-culture system Functional classification of the set of 78 of 108 deregulated genes in *CDKN2A* mutants are indicated in Fig.1a Up-regulated genes and Fig.1b Down-regulated genes. T. Factors (transcription factors), Immune modulation & IFN (Immunomodulation and interferon related genes); Cell. Components and metabolism (Cellular components and metabolism), Diff & Melanogenesis (differentiation and melanogenesis)

**Figure 2:**
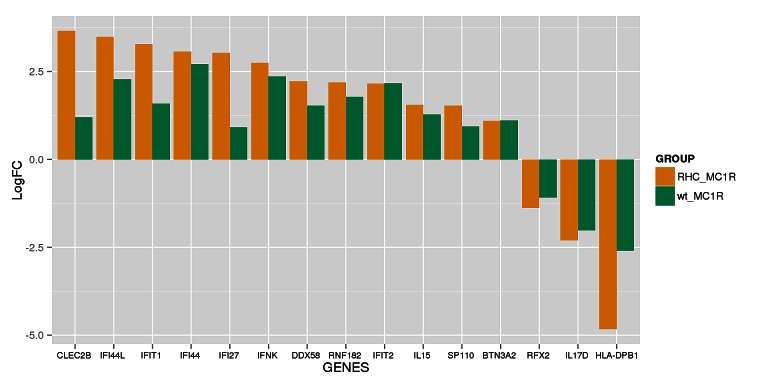
Set of immunomodulation and interferon response deregulated in mutant CDKN2A co-cultures The logarithm of the Fold change (LogFC) for the set of genes is indicated for wild-type *MC1R* co-cultures (wt_MC1R; green) and for Red Hair Colour *MC1R* variants co-cultures (RHC_MC1R; yellow).

Class comparison generated a signature of 108 unique genes that significantly discriminated between *CDKN2A* mutation and non-mutation (p-value <0.0001, listed in Table S1). Functional categorization of those transcripts belonging to known genes (Figure 1a, Figure 1b) showed that the mutant *CDKN2A* signature included deregulation of a vast number of genes involved in cell component and metabolism (37% up-regulated and 42% down-regulated,). Notably, immunomodulation and interferon response genes represented the second most prevalent subset of up-regulated transcripts (24%) and third down-regulated subgroup (13%) which may reflect a constitutively altered cytokine and chemokine profile in mutant cells. The set of deregulated genes involved in immunomodulation and interferon response includes: *CLEC2B, IFI44L, IFIT1, IFI44, IFI27, IFNK, DDX58, RNF182, IFIT2, IL15, SP110, BTN3A2, RFX2, IL17D* and *HLA-DPB1* (Figure 2). The molecular profile also included deregulation of genes which may confer a phenotype with the capability for malignant transformation since they are involved in cell adhesion, cell growth and proliferation. Over-expression of apoptosis related genes *CASP3*, *XAF1* and *SAMD9* was also detected. Transcription factors such as *CEBPZ, LEF1, MACC1, TBX1, ZNF404* and *ZNF8,* epidermal differentiation and melanogenesis genes such as *KRT2*, *TYPR1, LIMS3,* *EEA1* and *MFI2* were also components of the mutant *CDKN2A* signature.

Further examination by functional enrichment analysis revealed an over-representation of down-regulated genes from the Notch signaling pathway (hsa_04330; p=0.038) in mutant *CDKN2A* skin cells including *NCOR2, PSEN, DLLC1, DVL3, NOTCH3, RFNG* and the co-activator *MAML1* which controls the growth-promoting effect of Notch1.

### Impact of non-functional *MC1R* gene on Global Transcript Profiles of skin primary cultures

*MC1R* analysis was carried out following the same workflow used in the *CDKN2A* analysis. Whole genome expression profiling displayed 3570 transcripts differentially expressed in RHC variant (A1 and A2) vs. wild-type MC1R skin cells (B1 and B2). Specifically, 54% of transcripts (1954/3570) were up-regulated while 46% (1616/3570) were down-regulated. By class comparison, 159 genes showed highly statistically significant differences (p < 0.0000001) (listed in Table S2). RHC skin cells showed a down-regulation of a subset of genes categorized as melanocyte differentiation and pigmentation genes. The transcription of most of these genes is regulated directly via the *MC1R* signaling pathway, or indirectly through keratinocyte differentiation genes *(TYRP1, MLANA, TYR, CRNN, PADI1, GDF15, MLPH, KIT, INHBB, DAB2, KLK6, FOXC1,* and *NDRG4)*. Furthermore, RHC variant skin cells exhibited up-regulation of DNA damage and/or DNA repair response genes (*BUB1, CHEK1, POLQ, RAD51AP1, RAD54B, RAD51C, BLM, UBE2T, GINS2, EXO1, FAM33A,* *PTTG2, PTTG1, RAD54L, DTL),* cell cycle and proliferation genes *(CDKN3, CDC25, CDK1, CCNA2, CDC20,* *CCNB2, CCNB1, CKS, CDCA3, PLK1,* *PRIM, CDCA8, GMNN, CDC6, VRK1, CKS1B, RFC4, CDC45, CENPN, HAUS8, PSRC, MCM6, MELK, AURKA, NCAPG)* and microtubule-based motors kinesin superfamily genes *(KIF11, KIF15, KIF2C, KIF4A,KIF23).*

**Figure 3:**
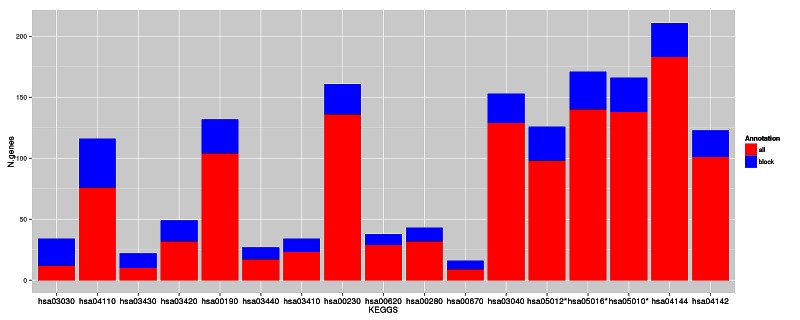
Deregulated pathways in RHC MC1R melanocyte-keratinocyte co-culture system In blue are the number of total genes annotated within the pathway, in red are the number of deregulated genes in the RHC *MC1R* co-cultures. The KEGG term is indicated for each pathway (axis X). * Pathways related to neurodegenerative diseases.

The functional enrichment analysis showed a considerably higher number of altered pathways in non-functional *MC1R* cells than in *CDKN2A* mutant cells. The RHC skin cells displayed over-expression of genes playing a role in major DNA repair and cell homeostasis pathways such as DNA replication, cell cycle and also oxidative phosphorylation which leads to production of reactive oxygen species (Figure 3 and Table 2). The lysosome and endocytosis pathways, which are intrinsically related to melanosome transfer from melanocytes to keratinocytes, were over-represented in the set of down-regulated transcripts as a direct consequence of low pigment production in RHC skin cells. Interestingly, RHC skin cells also exhibited an up-regulation of genes involved in neurodegenerative disorders such as Parkinson´s disease (hsa05012), Huntington´s disease (hsa5016) and Alzheimer´s disease (hsa05010). Although most of the genes were involved in several of these pathways, some of them were restricted to Huntington’s disease (*AP2B1, CREB3L4, POLR2H* and *SOD1*), to Alzheimer´s disease (*NAE1*, *FAS*) or to Parkinson’s disease (*PARK7).*

**Table 2:** Deregulated pathways in RHC MC1R melanocyte-keratinocyte co-culture system.

KEGG Term	STATUS	Adj. P-Value
DNA replication (hsa03030)	UP	3.83E-14
Cell cycle (hsa04110)	UP	7.87E-14
Mismatch repair (hsa 03430)	UP	1.56E-06
Nucleotide excision repair (hsa03420)	UP	7.01E-06
Oxidative phosphorylation (hsa00190)	UP	6.91E-05
Homologous recombination (hsa03440)	UP	6.98E-04
Base excision repair (hsa03410)	UP	1.07E-03
Purine metabolism (hsa00230)	UP	1.80E-02
Pyruvate metabolism (hsa00620)	UP	3.47E-02
Valine. leucine and isoleucine degradation (hsa00280)	UP	8.13E-03
One carbon pool by folate (hsa00670)	UP	2.69E-03
Spliceosome (hsa03040)	UP	1.80E-02
Parkinson's disease (hsa05012)	UP	3.10E-05
Hugtington's disease (hsa05016)	UP	5.25E-04
Alzheimer's disease (hsa 05010)	UP	3.03E-03
Endocytosis (hsa04144)	DOWN	3.83E-02
Lysosome (hsa04142)	DOWN	4.70E-03

The KEGG term is indicated for each pathway (X-axis). Status: UP: pathway overrepresented in the group of up-regulated transcripts in RHC MC1R; DOWN: pathway over-represented in the group of down-regulated transcripts in RHC MC1R.

Besides the lack of functionality caused by p.R151C and p.R160W variants, we also analyzed a possible effect on *MC1R* levels, by the relative quantification of *MC1R* expression. We observed a statistically significant reduction in *MC1R* expression in RHC skin cells (ddCt=0.06±0.02) compared to wild-type *MC1R* skin cells (ddCt=0.50±0.07; P-value<0.0001). Furthermore, the expression of *FARP1, SLFN11, GFPT2, COL5A3, TYRP1, LEF1, KRT2, ST6GALNAC3, MLANA, MSMB, SILV, A2M,* and *ALOX5* was evaluated by RT-PCR confirming the results obtained in the array (data not shown).

**Figure 4:**
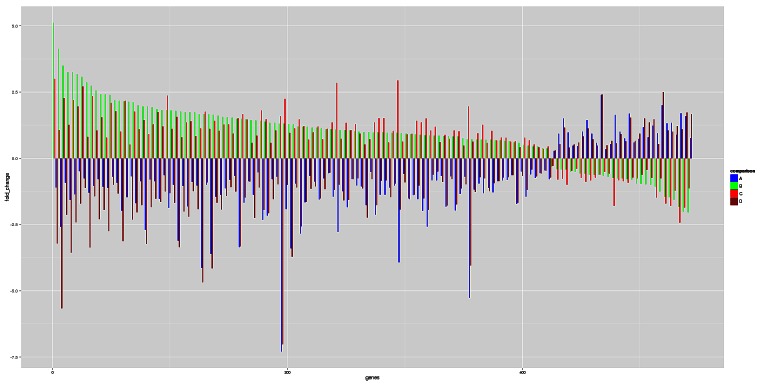
Genes inversely expressed in mutant CDKN2A and RHC MC1R 136 genes were found inversely expressed between mutant *CDKN2A* and RHC *MC1R* genes (listed in Table S3). A: RHC *MC1R* vs wt *MC1R* (both mutant *CDKN2A*); B: mutant *CDKN2A* vs wt *CDKN2A* (both RHC *MC1R*); C: mutant *CDKN2A* vs wt *CDKN2A* (both wt *MC1R*); D: RHC MC1R vs wt MC1R (both wt *CDKN2A*).

We further examined the overlap between genes differentially expressed in both mutant *CDKN2A* (n=1536) and in non functional MC1R cells (n=3570). A total of 485 genes were altered in the same fashion in both mutant *CDKN2A* and non functional *MC1R* cells and the expression of 136 genes was found inversely correlated between both groups. Of these 136 genes (listed in Table S3), 30 were up-regulated in mutant *CDKN2A* and down-regulated in non functional MC1R cells and 106 genes were down-regulated in mutant *CDKN2A* and up-regulated in non functional *MC1R* cells (Figure 4). The functional analysis of these 136 genes did not reveal over-representation of any specific pathway.

### *In silico* model evaluation

**Figure 5:**
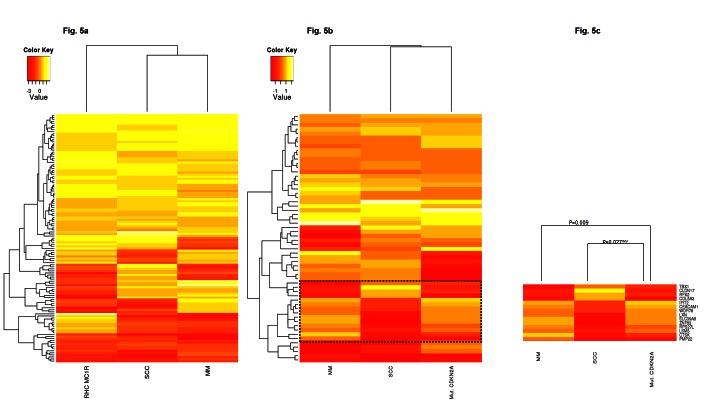
In-silico model evaluation Comparison of the expression patterns detected in our study to those observed in Squamous cell Carcinomas (SSC; GSE2503 dataset) and Melanomas (MM; GSE12391 dataset). **Fig.** 5A: Heatmap *MC1R* expression pattern showed a positive correlation among datasets (p-value <0.005). **Fig.** 5B: Heatmap *CDKN2A* expression pattern showed a positive correlation among datasets (p-value <0.06). **Fig.** 5C: Subset of genes with an inverse expression pattern between mutant *CDKN2A* skin cells and SSC and MM. ^INV^: inversely correlated pattern.

We hypothesized that if the altered gene signatures observed in non-lesional skin from *CDKN2A* mutation or RHC *MC1R* variant carriers are critical for malignant transformation, these patterns will be maintained across the carcinogenic process. To support our hypothesis, the expression pattern of the most deregulated genes in mutant *CDKN2A* or RHC *MC1R* variant skin cells was compared to their expression observed in two previously published skin cancer datasets. The GSE2503 dataset contained the whole genome expression of five squamous cell carcinomas (SCCs) and six healthy skin samples[35] and the GSE12391 dataset included 23 melanomas (CMs) at different stages (radial growth phase or vertical growth phase) and 18 common nevi[36]. Due to differences in the array platforms used among the studies, only the expression of genes present in all datasets was evaluated (Figure 5). Thus, *MC1R* expression pattern analyses included 76.1% (121/159) of the most deregulated genes. Primary co-cultures with the non-functional *MC1R* gene showed a statistically significant expression pattern (p-value <0.005) similar to those detected in SCCs (Pearson correlation=0.44) and CMs (Pearson correlation=0.25) (Figure 5a).

Because of platform disparities, the mutant *CDKN2A* expression pattern was evaluated analyzing 53.7% of the most deregulated transcripts (58/108). The expression pattern correlated to those observed in SCCs (Pearson correlation=0.35) and CMs (Pearson correlation=0.28) showing a statistical tendency among datasets (p-value <0.06), in which immunomodulation and interferon response genes were also up-regulated (*SP110*, *BTN3A2, IL15, IFIT2, IFI44, IFIT1, IFI44L* and *CLEC2B;* Figure 5b). A correlated expression pattern was found within a subset of genes between CMs and mutant *CDKN2A* skin cells (Pearson Correlation=0.66, P-value = 0.009; Figure 5c). Remarkably, this subset of genes presented an expression pattern that inversely correlated between SCCs and CMs (Pearson correlation= -0.88, p-value<0.001) and also between SCCs and mutant *CDKN2A* skin cells (Pearson correlation= -0.58, p-value=0.027). Specifically, the expression pattern of *TBX1, CLDN17, RFX2, COL5A3, IFIT2, CEACAM1, WDR78, LXN, SLC39A8, ZNF83, RPS27L, LSM3, CTSK*, and *PMP22* was exclusively correlated to MM and mutant *CDKN2A* MM prone skin cells, and inversely with NMSC.

## DISCUSSION

The aim of the study was to determine the constitutional effect of germline *CDKN2A* mutations or *MC1R* variants in skin cancer, in order to identify early critical molecular targets implicated in the disease. To do so we analyzed co-cultured melanocyte-keratinocyte systems derived from two siblings belonging to two melanoma prone families with a founder *CDKN2A* mutation and/or carrying non-functional *MC1R* alleles to compare global gene expression profiles.

To date, *CDKN2A* is the major gene responsible for increased melanoma susceptibility in high-risk pedigrees. Germline high-penetrance mutations are found in 10% of melanoma prone families and somatic *CDKN2A* alterations are also recurrent events in primary melanomas[37] and melanoma cell lines[38]. Alterations in gene expression in as yet phenotypically normal cells but bearing single-hit mutations in tumor suppressor genes appear to be the earliest molecular change during cancer development[39].

Altered baseline expression signatures associated with *CDKN2*A mutations in cultured normal skin fibroblasts from familial melanoma patients have already been reported [40]. However, the transcriptome deregulation within keratinocytes and melanocytes where cutaneous carcinomas and melanomas arise have not been previously assessed. We found that transcriptome signatures were altered by single hit *CDKN2A* mutations in co-cultured melanocyte-keratinocyte systems. *CDKN2A* mutant cells exhibit mainly baseline differences in genes related to cell components, metabolism and immune response. Melanoma cells are addicted to oncogene-driven energy production which can be mediated by somatic *BRAF* mutations [41], our findings indicate that non transformed skin cells already have a deregulated metabolic profiling which may be necessary for the skin cancer initiation.

Previous studies suggest that genetic variation of some cytokines or their receptors influence melanoma susceptibility [42, 43] and even modify the risk in melanoma-prone families with *CDKN2A* mutations [44]. Consistent with this hypothesis, our data reflects the important role of innate immunity and immune response pathways which may be deregulated in early steps of melanoma development. Skin cells also exhibited deregulation of epidermal differentiation and melanogenesis genes. Thus, we found down-regulation of the *TYRP1* gene, a melanocyte differentiation marker and over expression of *MFI2* gene, a cell-surface glycoprotein playing a role in melanoma cell proliferation and tumorigenesis [45] and *EEA1* gene, a marker of early endosomes [46]. Interestingly, *EEA1* interacts with *MFI2* regulating endosome fusion and trafficking [47]. Early endosomes are direct precursors of melanosomes, which are the specialized organelles for the biosynthesis and storage of melanins[46]. These findings may indicate that constitutive disorganized melanosomal structures and changes in pigment production may favor malignant transformation which in turn, could be related to autoimmune response deregulation [48, 49].

Via functional analyses, we observed a down-regulation of the Notch signaling pathway in *CDKN2A* mutant cells. This pathway is essential in epidermal-melanocyte interactions [50, 51] and recent evidence suggests Notch pathway as a link between the control of epidermal differentiation and proliferation and skin homeostasis [52, 53]. Notch signaling has a dual action (as oncogene or as tumor suppressor) in skin cancer. While, up-regulation of the Notch pathway is observed in CMs or in SCCs at sun-protected sites, it is down-regulated in UV-related solar keratoses SCCs or in common melanocytic nevi (reviewed in Panelos and Massi, 2009 [54]). A high melanocytic nevi count is the strongest known risk factor for melanoma[55] and a potent predictor of mutant *CDKN2A* gene carrier status[56]. Thus, the baseline down-regulation of the Notch signaling pathway in *CDKN2A* mutation carriers may proffer a melanocyte proliferation advantage, which can trigger common nevi development in human skin.

The pigmentation related gene *MC1R* acts as a moderate melanoma risk gene and variants in this gene are modifying factors for melanoma risk in *CDKN2A* mutation carriers [21-23]. Furthermore, *MC1R* variants have also been clearly associated with elevated NMSC susceptibility [19, 20]. We observed that the number of genes differentially expressed in non-functional *MC1R* was considerably higher than in mutant *CDKN2A*. Both p.R151C and p.R160W alleles halted *MC1R* activity and also induced an altered cell surface molecule expression due to impaired export traffic [57] that could lead to a reduction in *MC1R* gene expression levels as observed in our study. We found that RHC *MC1R* skins cells differ from wild-type in the deregulation of genes involved in key physiological processes such as differentiation, cell adhesion and cell cycle progression which may be directly related to the etiology of skin cancer. We found that in non-functional *MC1R* cells, pathways associated with increased DNA damage without UV radiation exposure were up-regulated, indicating that RHC variants contribute to cancer risk in humans increasing the DNA damage mediated by intrinsic UV-independent mechanisms as recently observed in mice models [58]. Notably, RHC *MC1R* skin cells also showed up-regulated pathways related to neurodegenerative diseases. The *MC1R* gene is expressed in cells of the nervous system and its activation decreases oxidative stress and has anti-inflammatory and immunomodulatory effects [59]. Co-occurrence of Parkinson’s disease (PD) and CMs [60, 61] has been reported in epidemiological studies. The diagnosis of CM is associated with an approximate 50% increased risk of subsequent PD[61] development whereas individuals with PD have a two-fold increase in risk of subsequent CM[62] development. Previous data suggests that this relation is associated with pigment-related genes[63] rather than Parkinson’s-related genes[64, 65]. Our study further supports that *MC1R* is involved in the cross-link between both diseases. Thus, *MC1R* variants may increase the oxidative damage in brain cells and deregulate inflammatory processes which consequently, increase the susceptibility to neurodegenerative disorders.

Previous studies showed that many of the alterations associated with transcriptome and proteome signatures of as yet phenotypically normal cells bearing inherited alterations are also present in the corresponding form of cancer [39]. Thus, in our study, expression patterns detected in MM prone cells from non-lesional areas of the skin were compared with those observed in skin cancer tumors (SCCs and CMs) [35, 36]. Although differences in the experimental designs among studies (normal skin cells *vs* tumoral tissue or cultured cells *vs* fresh-frozen tissue) may interfere in the analysis, the expression patterns detected in our study correlate to those observed in skin tumors. Interestingly, the *MC1R* expression pattern was more similar among studies than the *CDKN2A* pattern (p-value<0.005 and p-value<0.06, respectively). These differences are caused in part by a subset of genes showing a converse pattern which allows us to distinguish between SSCs versus CMs and mutant *CDKN2A* skin cells (MM prone cells). Accordingly, previous studies have detected the inverse expression of two genes from this subset (*CEACAM1* and *CTSK)* between SSCs and MMs [66, 67]. Such differences may underlie the epidemiological differences observed between *MC1R* and *CDKN2A* and skin cancer susceptibility since *MC1R* polymorphisms are involved in both CMM and NMSC susceptibility while *CDKN2A* mutations are closely related to CM susceptibility rather than SCC susceptibility.

In conclusion, we have identified baseline expression signatures in skin cells carrying germline *CDKN2A* mutations and RHC *MC1R* variants which are maintained in skin tumors (melanoma and squamous cell carcinomas). The study identified a large catalogue of genes in *CDKN2A* mutant skin cells that are closely related to skin cancer, highlighting the role of genes involved in immune response, in melanosome biogenesis and the Notch signaling pathway Also, our data indicates that non functional *MC1R* variants promote DNA damage by intrinsic UV-independent mechanisms in human skin cells. Furthermore, our study revealed a role for *MC1R* in the susceptibility to neurodegenerative diseases which may be related to its role in oxidative stress and inflammatory processes.

## METHODS

### Study Design

To detect the effect of *CDKN2A* and *MC1R* genes, two melanoma families (A and B) were selected (Table I). The most frequent mutation, p.G101W, resulting from a common ancestor in Mediterranean pedigrees, was chosen in relation to the *CDKN2A* status. Regarding *MC1R* status, the presence of two red hair variants in each individual (RHC: p.R151C and p.R160W) was analyzed. To reduce the effect of intra-individual variability, two siblings from each family were selected, resulting in four different extreme genotypic conditions: a carrier of variants in both genes (sample A1), an individual without variants in any gene (sample B2) and two carriers of variants in one of these genes (samples A2 and B1), respectively.

The study was approved by the IRB and signed informed consents were obtained from all individuals.

### Primary keratinocyte and melanocyte co-culture

Skin biopsies from non-lesional areas were taken and human keratinocytes and melanocytes were obtained by mechanical fragmentation and enzymatic digestion [68]. Briefly, skin biopsy fragments were digested with collagenase type I solution (2mg/ml) (Sigma–Aldrich, Gillingham, UK) for 90 minutes. The collagenase solution was then completely eliminated and the remaining skin biopsy was introduced into a mixture of 0.05% trypsin /0.02% EDTA (T/E) (Sigma). Every 30 min, T/E was changed for a fresh T/E mixture. Collected T/E was inactivated with serum containing culture medium and was centrifuged at 1400 rpm for 10 minutes. Primary keratinocytes and melanocytes obtained by this method were cultured on a feeder layer of lethally irradiated (X-ray; 50 Gy) 3T3-J2 cells (a gift from Dr J. Garlick, SUNY), as previously described [69, 70]. The seeding media was a 3:1 mixture of Dulbecco’s Modified Eagle Medium (DMEM) (GIBCO-BRL, Barcelona, Spain) and HAM’S F12 (GIBCO-BRL) containing 10% FCS, 0.1 nM choleric toxin, 2 nM T3, 5 μg/mL insulin, and 0.4 μg/mL hydrocortisone. Cells were cultured at 37°C in a humid atmosphere containing 5% CO2. The culture medium was changed every two days. This co-culture system has been extensively proven to preserve stemness of keratinocytes[69-71] as well as to maintain the physiological melanocyte:keratinocyte ratio (1:40)[72, 73]. Moreover, by using the skin cells from this type of co-culture, the donor’s phenotypic pigmentation and UV-response features are preserved *in vivo* on a humanized skin mouse model[74].

### RNA extraction

Total RNA isolation from primary cultures on passage 3-4 was performed using the Trizol extraction method (Invitrogen Life Technologies, Carlsbad, CA) followed by purification in commercial columns (Qiagen, Valencia, CA). Total isolated RNA was further purified using an RNeasy kit (Qiagen, Valencia, CA). RNA concentration was determined using a NanoDrop Spectrophotometer (Thermo Scientific) and integrity of the RNA was verified by Bioanalyzer 2100 (Agilent, USA). The RNA integrity number was in all cases higher than 8.

### Expression array

Analysis of global expression was performed using the Whole Human Genome (4x44k) Oligo Microarray kit (G4112F, Agilent, US). The microarray contains probes from over 41,000 unique human genes and transcripts, all with public OMIM annotations (RefSeq, Goldenpath, Ensembl, Unigene Human Genome and GenBank databases). Overall, 50 ng of RNA were labeled using Low input Quickamp Labeling kit (Agilent, US). In all samples 10 commercial controls probes were added in order to standardize the results (RNA Spike-in kit, one color, Agilent, US). The arrays were scanned using the DNA Microarray Scanner G2565CA (Agilent, US). Finally, Feature Extraction Software (FES, Agilent, USA) was used both to perform the quality control process and to extract the information. Three replicates from each primary culture were analyzed.

### Quantitative real-time reverse transcriptase polymerase chain reaction (qRT-PCR) of selected genes

To confirm the microarray results, the expression of 13 genes (*FARP1, SLFN11, GFPT2, COL5A3, TYRP1, LEF1, KRT2, ST6GALNAC3, MLANA, MSMB, SILV, A2M,* and *ALOX5*) was validated by RT-PCR. Also, *MC1R* expression was evaluated. The *ACTB* gene was used for normalization. RNA derived from donors’ healthy skin was used as a calibrator in each reaction. A total of 200 ng of RNA was retrotranscribed to cDNA using Taqman PCR Core Reagent kit (Roche Applied Science, Penzbergf, Germany). Real-time PCR was performed using Taqman Universal PCR master Mix (Roche Applied Science, Penzbergf, Germany). Reaction was performed in an ABI Step One plus RT-PCR sequence detection instrument (Applied Biosystems, CA, US). Primer design and gene-specific probe selection were carried out by the Universal Probe Library software (UPL, Roche, Mannheim, Germany).

Data was evaluated using the relative quantification method of ddCt[75]. Expression values were evaluated by T-test for equality means using the SPSS 17.0. P-values less than or equal to 0.05 were considered statistically significant.

### Microarray data and statistical analyses

The Agilent Processed Signal was standardized across arrays using quantile normalization[76]. Differential gene expression analysis was carried out using the limma package from Bioconductor. Multiple testing adjustments of p-values were performed according to Benjamini and Hochberg methodology[77].

Gene set analysis was carried out for the Kyoto Encyclopedia of Genes and Genomes pathways (KEGG) using FatiScan[78] in Babelomics[79]. This tool detects significantly up- or down-regulated blocks of functionally related genes in lists of genes ordered by different criteria such as differential expression, KEGG pathways and others. The core of the method is based on an algorithm to test whether a set of genes, labeled with terms (biological information), contain significant enrichments of one or several of these terms with respect to another set of reference genes. FatiScan uses a Fisher’s exact test for 2×2 contingency tables for comparing two groups of genes and extracting a list of KEGG terms whose distribution among the groups is significantly different. Given that many KEGG terms are simultaneously tested, the results of the test are corrected for multiple testing to obtain an adjusted p-value. FatiScan returns adjusted p-values based on the False Discovery Rate (FDR) method[77]. KEGG Pathways for the genes in the microarray where taken from the KEGG web.

In order to gain insight into the co-regulation patterns on transcript expression profiles, data from our microarray experiments were clustered together with expression data obtained from two previous experiments focused on NMSC (GSE2503 dataset[35]) and MM (GSE12391 dataset[36]). Raw data were obtained from the Gene Expression Omnibus. For each dataset we analyzed the differential expression and determined the fold-changes. We selected two subsets: genes differentially expressed in our study for the *MC1R* comparison (RHC alleles vs. wild-type *MC1R*) and a second group of genes differentially expressed in our study for the *CDKN2A* comparison (p.G101W vs. wild-type *CDKN2A)*. In each scenario, fold-changes were normalized using the quantile method[76] to compare differential expression results in all experiments. We performed a hierarchical clustering analysis of differentially expressed transcripts and the graphical representation showed the relationship between experiments. The correlation analysis quantified the linear relationship between the different studies m

### Supplemental Data Description

Supplemental Data includes two tables with the list of the most deregulated genes associated with *CDKN2A* mutations or *MC1R* variants; and one table containing the list of genes inversely deregulated between CDKN2A and MC1R.

## SUPPLEMENTARY TABLES



## Web Resources

Bioconductor (http://www.bioconductor.org)

Babelomics (http://babelomics.bioinfo.cipf.es/)

Kyoto Encyclopedia of Genes and Genomes pathways (KEGG) (http://www.genome.jp/kegg/)

Ensembl 56 release (http://www.ensembl.org)

Gene Expression Omnibus (http://www.ncbi.nlm.nih.gov/geo/)

Accession Numbers The GEO accession number for the expression microarray data reported in this paper is GSE44805.

http://www.ncbi.nlm.nih.gov/geo/query/acc.cgi?token=ptorruosgomwqds&acc=GSE44805
