# Supplementary material for: Capturing the biological impact of CDKN2A and MC1R genes as an early predisposing event in melanoma and non melanoma skin cancer
Source: Oncotarget. 2013 Dec 16;5(6):1439–51. doi: 10.18632/oncotarget.1444 (PMC4039222; doi:10.18632/oncotarget.1444)
Supplement: Supplementary file 1 [file oncotarget-05-1439-s001.pdf]

Supplementary Material:

Table S1: List of the most significant deregulated transcripts regarding *CDKN2A* status (p.G101W vs wild-type), adjusted P-value< 0.0001. Only genes identified in both *MC1R* wild-type and *MC1R* RHC variant carriers are included (N=108)
Abbreviations: FC: Fold Change; P-value: adjusted P-Value of such comparison; wt: wild-type.
*Statistical values of mutant *CDKN2A* vs wild-type in *MC1R* RHC skin cells (individual A1 vs individual A2).
**Statistical values of mutant *CDKN2A* vs wild-type in wild-type *MC1R* skin cells (individual B1 vs individual B2)

Gene ID	Gene Name	*FC* in *MC1R RHC* *	P-value in *MC1R RHC* *	*FC* in wt *MC1R***	P-value in wt *MC1R***
NM_002121	*HLA-DPB1*	-4.83	4.05E-13	-2.60	5.79E-10
NM_005766	*FARP1*	-3.6	1.32E-11	-1.53	5.80E-07
NM_152270	*SLFN11*	-3.4	1.87E-06	-2.08	7.47E-07
NM_005110	*GFPT2*	-3.2	5.49E-06	-2.50	5.64E-05
NM_015719	*COL5A3*	-3.1	1.87E-06	-2.28	3.91E-05
NM_000550	*TYRP1*	-2.76	1.43E-06	-2.34	6.40E-06
NM_016269	*LEF1*	-2.69	1.73E-09	-1.64	6.46E-07
NM_000423	*KRT2*	-2.45	7.93E-05	-2.70	1.58E-05
NM_000304	*PMP22*	-2.41	9.22E-10	-1.80	3.50E-08
NM_152996	*ST6GALNAC3*	-2.38	1.38E-08	-1.44	4.65E-06
NM_138284	*IL17D*	-2.31	7.53E-06	-2.02	2.08E-05
AF086044	*unknown*	-2.23	1.74E-10	-3.42	5.23E-13
NM_198951	*TGM2*	-2.08	4.10E-06	-2.42	4.33E-07
NM_138375	*CABLES1*	-2.08	5.78E-09	-3.31	8.92E-12
NM_006426	*DPYSL4*	-2.03	3.01E-05	-3.92	6.81E-09
NM_032048	*EMILIN2*	-1.83	5.82E-06	-2.04	1.00E-06
AK074473	*unknown*	-1.83	6.09E-06	-2.43	1.29E-07
NM_001129	*AEBP1*	-1.81	2.43E-07	-1.40	3.91E-06
NM_014070	*unknown*	-1.78	2.71E-07	-1.31	8.02E-06
BC004343	*C21orf122*	-1.66	5.99E-06	-1.47	1.48E-05
NM_148897	*SDR9C7*	-1.63	3.91E-05	-1.83	6.51E-06
AF131834	*unknown*	-1.53	2.07E-06	-2.60	1.81E-09
NM_080647	*TBX1*	-1.53	4.46E-06	-1.21	3.80E-05
NM_012131	*CLDN17*	-1.47	4.17E-05	-1.79	2.57E-06
NM_000635	*RFX2*	-1.38	2.98E-06	-1.08	2.98E-05
ENST00000224809	*unknown*	-1.28	6.59E-06	-1.81	6.37E-08
NM_032534	*KRBA1*	-1.21	2.92E-06	-0.97	2.25E-05
NM_152359	*CPT1C*	-1.08	1.08E-05	-1.48	1.50E-07
NM_001077263	*TMPRSS13*	-0.87	4.43E-05	-2.81	9.51E-12
NM_000396	*CTSK*	-0.74	7.83E-05	-1.81	1.05E-09
NM_003798	*CTNNAL1*	0.71	6.15E-05	0.81	9.91E-06
NM_015920	*RPS27L*	0.77	4.43E-05	0.86	8.34E-06
NM_001015509	*unknown*	0.79	1.13E-05	0.68	3.46E-05
THC2657938	*unknown*	0.81	4.28E-05	2.08	2.37E-10
NM_000022	*ADA*	0.87	8.65E-06	1.08	3.92E-07
THC2698177	*unknown*	0.89	6.21E-05	1.75	1.38E-08
NM_005760	*CEBPZ*	0.90	3.14E-05	2.20	3.14E-10
NM_004346	*CASP3*	0.91	1.50E-05	1.31	1.23E-07
NM_004487	*GOLGB1*	0.95	5.49E-06	1.17	2.84E-07
BC027178	*PRPF40A*	0.99	8.74E-05	2.62	3.74E-10
NM_004280	*EEF1E1*	1.00	7.97E-05	1.06	2.48E-05
NM_004986	*KTN1*	1.04	2.90E-06	2.53	1.59E-11
ENST00000377156	*unknown*	1.07	1.97E-05	1.09	9.97E-06
NM_007047	*BTN3A2*	1.09	1.77E-05	1.11	9.31E-06

NM_020169	*LXN*	1.11	3.10E-05	1.97	1.97E-08
ENST00000306024	*LSM3*	1.12	2.45E-05	0.99	5.33E-05
NM_001033719	*ZNF404*	1.13	4.49E-05	2.13	1.58E-08
NM_016010	*FAM164A*	1.13	1.23E-05	0.99	3.29E-05
NM_001007234	*unknown*	1.15	1.63E-06	1.42	7.87E-08
NM_001012334	*MDK*	1.16	6.19E-05	1.20	2.48E-05
NM_181453	*GCC2*	1.19	6.48E-05	1.56	1.83E-06
NM_033514	*LIMS3*	1.20	8.85E-07	1.16	8.49E-07
NM_033316	*MFI2*	1.20	9.60E-05	1.87	3.91E-07
NM_002890	*RASA1*	1.22	1.18E-06	0.86	3.86E-05
NM_018300	*ZNF83*	1.23	5.73E-05	1.70	8.63E-07
NM_201612	*unknown*	1.23	1.19E-05	2.28	4.38E-09
NM_207014	*WDR78*	1.30	6.81E-05	1.50	8.56E-06
NM_002078	*GOLGA4*	1.31	4.03E-05	1.21	5.59E-05
AK075484	*SAMD4B*	1.31	1.52E-05	3.52	3.01E-11
NM_003566	*EEA1*	1.33	9.48E-05	2.36	7.47E-08
NM_144777	*SCEL*	1.33	5.55E-07	1.01	9.54E-06
CB050071	*unknown*	1.41	5.52E-05	1.53	1.31E-05
NM_022154	*SLC39A8*	1.43	8.09E-05	1.54	2.19E-05
CR603437	*unknown*	1.44	1.33E-06	1.35	1.75E-06
NM_018169	*C12orf35*	1.48	2.17E-05	2.02	3.71E-07
NM_207331	*unknown*	1.49	9.36E-06	1.28	3.07E-05
AB075826	*FAM171B*	1.49	5.31E-05	1.47	3.83E-05
NM_004510	*SP110*	1.53	1.66E-07	0.94	3.63E-05
NM_172174	*IL15*	1.55	5.30E-08	1.29	4.08E-07
NM_000888	*ITGB6*	1.55	3.99E-05	2.52	8.05E-08
NM_005059	*RLN2*	1.58	1.97E-05	1.38	5.37E-05
NM_025114	*CEP290*	1.67	1.90E-05	2.70	3.88E-08
AK096536	*unknown*	1.70	3.16E-06	1.36	2.52E-05
THC2683448	*unknown*	1.72	2.01E-06	1.91	3.87E-07
NM_001013728	*unknown*	1.74	1.34E-05	2.23	4.47E-07
BC029919	*unknown*	1.78	4.62E-06	1.40	4.30E-05
NM_001712	*CEACAM1*	1.80	1.35E-06	1.56	4.49E-06
NM_003020	*SCG5*	1.83	2.10E-08	2.37	5.14E-10
NM_004921	*unknown*	1.86	8.41E-05	1.74	9.87E-05
NM_018429	*BDP1*	1.90	1.48E-05	2.05	3.60E-06
THC2676635	*unknown*	1.93	2.93E-08	1.27	3.49E-06
NM_178445	*CCRL1*	1.99	1.68E-05	1.98	1.08E-05
THC2740317	*unknown*	2.04	7.85E-06	1.75	2.63E-05
NM_182762	*MACC1*	2.05	5.66E-09	0.89	8.55E-05
XR_015158	*unknown*	2.15	6.40E-10	1.91	2.32E-09
NM_001547	*IFIT2*	2.16	4.68E-08	2.17	3.56E-08
NM_152737	*RNF182*	2.19	1.05E-05	1.78	5.97E-05
NM_014314	*DDX58*	2.22	3.99E-08	1.53	2.66E-06
AK022351	*unknown*	2.26	4.01E-05	2.74	2.66E-06
THC2526509	*unknown*	2.26	6.31E-08	1.19	7.99E-05
NM_181501	*ITGA1*	2.38	1.07E-08	1.09	8.38E-05
NM_018284	*GBP3*	2.40	1.80E-07	2.10	6.58E-07
AI078143	*unknown*	2.60	2.11E-07	1.52	7.46E-05
THC2510656	*unknown*	2.62	8.54E-06	2.91	1.51E-06
THC2549494	*unknown*	2.72	4.74E-06	2.48	8.65E-06
NM_020124	*IFNK*	2.74	7.98E-07	2.36	3.02E-06
NM_152703	*SAMD9L*	2.98	8.72E-06	2.94	6.15E-06
NM_005532	*IFI27*	3.03	3.42E-11	0.92	5.22E-05
NM_017654	*SAMD9*	3.04	1.08E-07	1.98	1.26E-05
NM_006417	*IFI44*	3.07	3.28E-12	2.71	7.74E-12
THC2679528	*unknown*	3.18	4.40E-07	2.89	9.26E-07
AK023743	*unknown*	3.18	1.52E-10	1.95	7.56E-08
NM_001548	*IFIT1*	3.28	6.99E-11	1.59	5.54E-07
BF213738	*unknown*	3.46	3.06E-11	1.34	2.66E-06
NM_006820	*IFI44L*	3.49	6.99E-11	2.28	1.49E-08
D89479	*SULT1B1*	3.56	1.70E-08	3.24	4.36E-08

NM_005127	*CLEC2B*	3.66	1.61E-13	1.20	1.33E-07
NM_017523	*XAF1*	5.11	1.98E-08	3.00	9.40E-06

Table S2: List of the most significant deregulated transcripts regarding *MC1R* status (p. R106W and p.R151C vs wild-type) identified in both comparisons.
Adjusted P-value < 0.0000001. Only genes identified in both analyses are included (N=159)
Abbreviations: FC: Fold Change; P-value: adjusted P-Value of such comparison; wt: wild-type.
*Statistical values of *MC1R* RHC vs wild-type in mutant *CDKN2A* skin cell (individual A1 vs individual B1).
**Statistical values of *MC1R* RHC vs wild-type in wild-type *CDKN2A* skin cell (individual A2 vs individual B2).

Gene ID	Gene Name	FC in mutant *CDKN2A**	P-value in mutant *CDKN2A**	FC in wt *CDKN2A***	P-value in wt *CDKN2A***
NM_005672	*PSCA*	-7.30	2.39E-12	-7.02	1.20E-12
NM_000550	*TYRP1*	-7.24	2.85E-12	-6.82	2.14E-12
NM_000095	*COMP*	-6.79	3.34E-13	-5.08	2.95E-12
NM_005511	*MLANA*	-5.74	2.51E-09	-6.96	7.05E-11
NM_000372	*TYR.TYRL*	-5.59	1.29E-12	-7.19	1.62E-14
NM_002443	*MSMB*	-5.30	1.03E-14	-4.05	1.47E-13
NM_006928	*SILV*	-4.79	1.66E-08	-6.05	3.02E-10
NM_000166	*GJB1*	-4.72	2.43E-10	-2.76	9.95E-08
NM_000014	*A2M*	-4.52	3.06E-08	-7.21	2.40E-11
NM_000698	*ALOX5*	-4.17	3.63E-11	-2.19	6.05E-08
NM_016190	*CRNN*	-4.14	8.50E-10	-4.68	5.89E-11
THC2711870	*unknown*	-3.98	8.16E-09	-6.00	1.27E-11
THC2675163	*unknown*	-3.73	6.81E-12	-6.37	5.96E-15
NM_032588	*TRIM63*	-3.70	6.23E-10	-5.36	1.70E-12
NM_004750	*CRLF1*	-3.70	1.31E-10	-2.14	6.30E-08
NM_013358	*PADI1*	-3.61	1.08E-08	-4.16	6.97E-10
ENST00000366930	*TGFB2*	-3.56	3.31E-10	-2.27	4.78E-08
NM_004864	*GDF15*	-3.31	8.19E-11	-4.23	1.09E-12
NM_020182	*PMEPA1*	-3.18	2.39E-12	-1.98	4.44E-10
NM_001712	*CEACAM1*	-3.12	6.03E-10	-3.36	7.97E-11
NM_005512	*LRRC32*	-3.11	8.78E-08	-3.21	2.37E-08
NM_144497	*AKAP12*	-3.10	8.04E-08	-2.91	7.23E-08
NM_130808	*CPNE4*	-2.97	4.58E-09	-2.69	6.33E-09
NM_001901	*CTGF*	-2.94	9.98E-10	-4.13	4.12E-12
NM_001039212	*unknown*	-2.87	9.12E-11	-3.14	9.05E-12
NM_024101	*MLPH*	-2.86	1.13E-08	-4.17	3.06E-11
ENST00000264554	*SHC2*	-2.80	1.77E-08	-2.97	3.32E-09
NM_000201	*ICAM1*	-2.75	2.83E-09	-2.45	4.99E-09
NM_000222	*KIT*	-2.75	6.97E-09	-5.47	3.15E-13
NM_201631	*TGM5*	-2.69	6.67E-09	-2.57	4.59E-09
NM_002193	*INHBB*	-2.61	3.47E-10	-2.46	2.87E-10
NM_001001547	*CD36*	-2.60	1.37E-08	-2.58	6.28E-09
NM_001945	*HBEGF*	-2.59	4.84E-08	-2.56	2.20E-08
NM_002463	*MX2*	-2.59	2.72E-09	-5.66	4.69E-14
NM_173198	*NR4A3*	-2.45	2.44E-10	-4.42	5.15E-14
NM_001343	*DAB2*	-2.42	2.64E-08	-3.27	1.92E-10
NM_138375	*CABLES1*	-2.23	1.35E-09	-3.46	1.49E-12
NM_001012964	*KLK6*	-2.19	2.71E-09	-4.55	8.27E-14
NM_016463	*CXXC5*	-2.10	3.00E-08	-2.26	4.70E-09
NM_001453	*FOXC1*	-1.94	1.02E-09	-1.72	2.04E-09
NM_003793	*CTSF*	-1.92	4.54E-08	-3.15	2.38E-11
NM_022910	*NDRG4*	-1.84	9.53E-09	-1.55	3.44E-08
NM_006989	*RASA4*	-1.67	7.49E-08	-2.15	1.08E-09
NM_145804	*ABTB2*	-1.45	7.91E-08	-2.40	3.58E-11

NM_003565	*ULK1*	-1.39	7.35E-08	-1.75	1.36E-09
NM_030912	*TRIM8*	-1.25	5.80E-08	-1.13	8.84E-08
NM_206836	*PECI*	1.33	6.12E-08	1.23	6.13E-08
NM_005915	*MCM6*	1.33	9.39E-08	1.69	1.62E-09
AY163812	*C17orf80*	1.34	6.62E-08	1.47	7.72E-09
CR600585	*unknown*	1.45	8.28E-09	1.35	7.88E-09
NM_033514	*LIMS3*	1.46	3.69E-08	1.42	2.13E-08
NM_016126	*HSPB11*	1.47	6.42E-08	1.48	2.29E-08
NM_001079525	*PAICS*	1.50	1.12E-08	2.03	7.45E-11
NM_016448	*DTL*	1.53	5.00E-09	1.85	1.41E-10
NM_003579	*RAD54L*	1.54	6.67E-09	1.87	1.86E-10
NM_032636	*PSRC1*	1.59	5.94E-08	1.67	1.21E-08
NM_033417	*HAUS8*	1.59	6.77E-08	1.71	1.05E-08
NM_018455	*CENPN*	1.61	2.25E-08	2.40	4.13E-11
NM_030771	*CCDC34*	1.64	3.02E-09	3.26	1.47E-13
NM_017868	*TTC12*	1.67	7.74E-10	2.18	7.74E-12
NM_003258	*TK1*	1.72	8.63E-08	1.83	1.53E-08
NM_003504	*CDC45L*	1.75	6.55E-09	1.99	4.57E-10
NM_002916	*RFC4*	1.76	1.32E-09	2.19	2.50E-11
ENST00000367003	*C1orf97*	1.77	2.25E-08	2.65	4.11E-11
NM_145231	*C14orf143*	1.84	1.04E-08	1.86	3.46E-09
NM_003798	*CTNNAL1*	1.85	2.44E-10	1.94	4.44E-11
NM_014317	*PDSS1*	1.86	3.58E-08	2.19	1.71E-09
NM_004219	*PTTG1*	1.87	9.03E-09	1.92	2.48E-09
NM_001159	*AOX1*	1.87	8.16E-09	2.23	3.05E-10
NM_018186	*C1orf112*	1.94	5.99E-09	1.94	2.12E-09
NM_001826	*CKS1B*	1.96	5.59E-09	2.00	1.55E-09
BX640843	*unknown*	1.99	5.94E-09	2.51	9.73E-11
NM_182620	*FAM33A*	2.01	1.76E-09	1.72	5.18E-09
NM_003686	*EXO1*	2.01	2.93E-10	2.19	3.22E-11
NM_004282	*BAG2*	2.02	9.45E-10	2.61	1.14E-11
NM_014051	*TMEM14A*	2.09	1.45E-08	2.14	4.46E-09
NM_016095	*GINS2*	2.12	7.82E-10	2.14	2.35E-10
NM_145014	*HYLS1*	2.13	5.04E-08	2.66	1.08E-09
NM_024037	*C1orf135*	2.13	8.41E-10	2.22	1.78E-10
NM_003384	*VRK1*	2.15	6.03E-10	2.37	5.77E-11
BC040051	*unknown*	2.15	5.79E-08	2.00	5.83E-08
NM_001254	*CDC6*	2.20	8.16E-08	2.57	4.42E-09
NM_014176	*UBE2T*	2.23	1.12E-11	2.18	5.39E-12
NM_203467	*PPIL5*	2.23	9.58E-10	1.82	5.22E-09
NM_000057	*BLM*	2.26	7.59E-08	2.85	1.45E-09
NM_024808	*C13orf34*	2.27	8.38E-08	2.54	7.74E-09
NM_006607	*PTTG2*	2.27	2.24E-10	1.92	7.20E-10
NM_001928	*CFD*	2.30	3.69E-08	2.45	6.65E-09
NM_015895	*GMNN*	2.31	1.29E-10	1.77	1.51E-09
NM_018101	*CDCA8*	2.34	7.82E-10	2.36	2.52E-10
AK027541	*unknown*	2.35	2.05E-08	2.34	8.84E-09
NM_001766	*CD1D*	2.36	6.03E-09	2.53	9.33E-10
NM_002876	*RAD51C*	2.37	4.47E-10	2.20	4.50E-10
NM_000946	*PRIM1*	2.38	2.14E-09	2.22	1.94E-09
NM_005030	*PLK1*	2.40	8.38E-08	2.41	3.33E-08
NM_031299	*CDCA3*	2.41	2.46E-09	2.09	6.11E-09
NM_012415	*RAD54B*	2.43	6.39E-08	2.34	4.24E-08
NM_006479	*RAD51AP1*	2.46	3.02E-08	3.80	3.48E-11
NM_138555	*KIF23*	2.48	8.16E-09	2.87	4.69E-10
NM_018193	*FANCI*	2.50	4.23E-11	2.38	2.70E-11
NM_014791	*MELK*	2.50	1.91E-09	2.50	7.39E-10
NM_175617	*MT1E*	2.52	6.20E-10	2.12	2.36E-09
NM_012177	*FBXO5*	2.54	1.32E-09	2.45	8.26E-10
NM_001012271	*BIRC5*	2.55	9.58E-10	2.63	2.35E-10
NM_001012267	*CENPP*	2.56	2.85E-12	1.76	1.78E-10
NM_001827	*CKS2*	2.57	1.36E-10	2.22	3.37E-10

BC044246	*TMEM200A*	2.57	7.02E-09	2.04	5.61E-08
NM_012310	*KIF4A*	2.57	3.81E-09	2.48	2.30E-09
NM_199420	*POLQ*	2.58	6.02E-08	3.45	5.20E-10
NM_001274	*CHEK1*	2.58	1.29E-10	3.00	5.77E-12
NM_001005414	*unknown*	2.62	6.03E-10	1.95	1.10E-08
NM_182751	*MCM10*	2.62	2.54E-08	3.10	1.09E-09
NM_001010897	*SERP2*	2.65	3.23E-09	2.27	9.59E-09
NM_001034	*RRM2*	2.67	1.05E-08	2.47	1.16E-08
NM_145018	*C11orf82*	2.71	1.68E-10	2.95	1.85E-11
NM_031966	*CCNB1*	2.74	2.63E-11	2.84	5.39E-12
NM_005059	*RLN2*	2.78	9.81E-09	2.58	1.05E-08
NM_006845	*KIF2C*	2.80	1.74E-09	2.78	7.12E-10
NM_001211	*BUB1B*	2.81	4.88E-08	3.45	1.32E-09
NM_198433	*AURKA*	2.85	1.71E-08	2.81	8.31E-09
NM_004701	*CCNB2*	2.92	2.85E-12	3.11	5.71E-13
NM_020242	*KIF15*	2.93	5.10E-09	4.62	4.38E-12
NM_004523	*KIF11*	2.96	1.80E-08	3.18	2.81E-09
NM_001255	*CDC20*	3.05	4.70E-09	2.92	3.13E-09
BC013418	*C13orf3*	3.10	3.10E-09	3.42	3.24E-10
AL834537	*CCDC150*	3.11	3.65E-08	4.61	7.45E-11
NM_004336	*BUB1*	3.13	2.15E-10	3.42	2.35E-11
NM_004117	*FKBP5*	3.14	4.53E-08	3.43	5.67E-09
NM_052913	*TMEM200A*	3.14	8.40E-08	2.96	7.23E-08
NM_001067	*TOP2A*	3.14	9.65E-11	3.93	1.70E-12
NM_005733	*KIF20A*	3.15	1.78E-11	2.96	1.38E-11
NM_001813	*CENPE*	3.18	6.03E-10	4.69	1.24E-12
NM_152515	*CKAP2L*	3.18	8.97E-08	3.74	4.49E-09
NM_001237	*CCNA2*	3.19	2.93E-10	3.25	8.24E-11
NM_018136	*ASPM*	3.20	1.92E-09	3.55	1.78E-10
NM_007280	*OIP5*	3.21	1.51E-11	2.87	2.37E-11
NM_138419	*FAM54A*	3.23	3.04E-11	3.07	2.01E-11
NM_020675	*SPC25*	3.26	1.59E-12	3.45	1.87E-13
NM_018492	*PBK*	3.28	3.26E-09	3.95	9.92E-11
NM_022346	*NCAPG*	3.29	5.95E-13	3.50	7.81E-14
NM_024094	*DSCC1*	3.31	3.47E-10	3.99	1.02E-11
NM_032117	*MND1*	3.35	6.71E-12	3.77	5.71E-13
NM_001809	*CENPA*	3.38	7.61E-11	3.90	3.72E-12
NM_001786	*CDC2*	3.38	2.39E-12	3.27	1.24E-12
NM_145697	*NUF2*	3.40	8.28E-09	4.81	3.22E-11
NM_021186	*ZP4*	3.49	1.03E-14	4.91	3.21E-17
NM_016343	*CENPF*	3.52	1.25E-09	2.85	7.88E-09
NM_001790	*CDC25C*	3.60	3.47E-08	3.67	1.06E-08
NM_018131	*CEP55*	3.63	4.13E-10	3.89	5.89E-11
NM_016195	*KIF20B*	3.68	4.08E-10	2.62	1.36E-08
NM_014750	*DLGAP5*	3.71	2.39E-12	4.17	1.87E-13
NM_005192	*CDKN3*	4.04	7.94E-08	4.03	3.28E-08
NM_018365	*MNS1*	4.09	5.55E-10	3.31	3.38E-09
NM_012484	*HMMR*	4.51	2.85E-12	5.25	1.87E-13
NM_080672	*PHACTR3*	4.53	8.83E-10	3.81	3.34E-09
NM_001956	*EDN2*	4.78	2.38E-09	4.67	1.19E-09
NM_153046	*TDRD9*	4.83	1.87E-12	4.59	1.08E-12
NM_032935	*MT4*	5.04	2.85E-12	5.54	4.26E-13
BC004565	*unknown*	5.05	1.16E-11	2.65	2.23E-08

Table S3: List of genes inversely deregulated between *CDKN2A* and *MC1R* analyses. Abbreviations: F.C: Fold Change; P-value: adjusted P-Value of such comparison; wt: wild-type.

		mutant *CDKN2A* vs wt *CDKN2A* (both RHC *MC1R*)		mutant *CDKN2A* vs wt *CDKN2A* (both wt *MC1R*)		RHC *MC1R* vs wt *MC1R* (both mutant *CDKN2A*)		RHC *MC1R* vs wt *MC1R* (both wt *CDKN2A*)	
**HGNC_symbol**	**Ensembl_link**	**log F.C**	**adj.p.value**	**log F.C**	**adj.p.value**	**log F.C**	**adj.p.value**	**log F.C**	**adj.p.value**
XAF1	NM_017523	5.11	1.98E-08	3.00	9.40E-06	-1.11	0.041921124	-3.22	1.59E-06
MX2	NM_002463	4.14	1.32E-11	1.06	0.000133758	-2.59	2.72E-09	-5.66	4.69E-14
IFI44L	NM_006820	3.49	6.99E-11	2.28	1.49E-08	-0.93	0.000400236	-2.14	9.59E-09
IFIT3	NM_001549	3.26	8.18E-09	1.26	0.00036059	-1.57	3.37E-05	-3.57	5.21E-10
CAPN14	AK092257	3.25	3.91E-05	2.20	0.00113099	-1.37	0.032844763	-2.42	0.0002558
	AK023743	3.18	1.52E-10	1.95	7.56E-08	-0.49	0.039372674	-1.71	1.27E-07
IFI44	NM_006417	3.07	3.28E-12	2.71	7.74E-12	-0.76	5.31E-05	-1.12	2.60E-07
KRT19	NM_002276	2.86	3.59E-10	0.81	0.000744197	-1.31	4.49E-06	-3.36	8.78E-12
IFNK	NM_020124	2.74	7.98E-07	2.36	3.02E-06	-1.04	0.007725554	-1.43	0.000326988
IFI6	NM_022873	2.56	3.45E-08	1.05	0.000601236	-0.79	0.00606156	-2.30	2.96E-08
BTN3A3	NM_006994	2.42	1.07E-05	1.55	0.000669858	-1.08	0.011856791	-1.95	3.31E-05
LMO2	NM_005574	2.41	1.43E-10	0.79	1.00E-04	-1.12	1.67E-06	-2.75	5.51E-12
GBP3	NM_018284	2.40	1.80E-07	2.10	6.58E-07	-0.70	0.020231435	-1.00	0.000961856
RNF182	NM_152737	2.19	1.05E-05	1.78	5.97E-05	-0.93	0.015597153	-1.33	0.000585284
RHOBTB3	NM_014899	2.17	1.04E-05	1.01	0.008209935	-1.98	1.59E-05	-3.14	2.33E-08
IFIT2	NM_001547	2.16	4.68E-08	2.17	3.56E-08	-1.48	3.08E-06	-1.47	1.46E-06
USP18	NM_017414	2.14	1.55E-08	0.52	0.018990885	-0.69	0.002714314	-2.31	1.21E-09
EPSTI1	NM_033255	2.12	3.99E-05	1.77	0.000160602	-1.70	0.0002321	-2.05	1.60E-05
	A_32_P53976	2.01	3.17E-05	1.10	0.005270514	-0.86	0.026819981	-1.77	3.55E-05
RSAD2	NM_080657	1.96	0.001691807	1.44	0.012103521	-2.71	4.60E-05	-3.23	2.96E-06
	AK023660	1.96	4.65E-08	0.92	0.000195518	-0.81	0.000669435	-1.85	2.23E-08
	THC2676635	1.93	2.93E-08	1.27	3.49E-06	-0.86	0.000221405	-1.52	1.43E-07
CLCA3	NM_004921	1.86	8.41E-05	1.74	9.87E-05	-1.54	0.000333218	-1.65	8.58E-05
PARP9	NM_031458	1.83	2.21E-06	1.21	0.000138412	-0.63	0.024137843	-1.26	4.46E-05
SCG5	NM_003020	1.83	2.10E-08	2.37	5.14E-10	-1.87	8.28E-09	-1.32	2.93E-07
HS3ST1	NM_005114	1.80	2.00E-05	1.12	0.001411596	-1.00	0.003458263	-1.69	1.14E-05
CEACAM1	NM_001712	1.80	1.35E-06	1.56	4.49E-06	-3.12	6.03E-10	-3.36	7.97E-11
OAS1	NM_002534	1.76	0.000121473	0.79	0.039773363	-1.04	0.007622258	-2.01	7.90E-06
	L06175	1.75	0.000889078	1.36	0.004950943	-1.82	0.000403092	-2.22	2.73E-05
	AK124281	1.75	5.68E-05	1.40	0.00033138	-0.90	0.011971445	-1.25	0.000538014
C5orf56	ENST00000378953	1.73	0.000157045	0.84	0.030463445	-1.03	0.008749111	-1.93	1.47E-05
CRNN	NM_016190	1.67	0.000105303	1.13	0.002501087	-4.14	8.50E-10	-4.68	5.89E-11

	AK025669	1.67	0.000315954	1.77	0.000112469	-1.00	0.013197622	-0.90	0.017900911
PADI1	NM_013358	1.66	0.000201315	1.12	0.004289502	-3.61	1.08E-08	-4.16	6.97E-10
	THC2640099	1.63	0.003962305	1.41	0.008167973	-1.45	0.006843443	-1.67	0.001407815
TNFSF10	NM_003810	1.61	3.76E-05	1.04	0.001525958	-1.37	0.000112711	-1.93	1.06E-06
F3	NM_001993	1.56	0.007308907	1.28	0.019400875	-1.14	0.039539591	-1.42	0.007227404
IL15	NM_172174	1.55	5.30E-08	1.29	4.08E-07	-0.82	5.92E-05	-1.09	1.10E-06
SP110	NM_004510	1.53	1.66E-07	0.94	3.63E-05	-0.67	0.000916414	-1.26	4.53E-07
BST2	NM_004335	1.49	0.00349873	1.52	0.00211498	-3.36	4.60E-07	-3.33	2.09E-07
CLCA4	NM_012128	1.49	0.001612926	1.67	0.000362464	-1.67	0.000354497	-1.48	0.000637245
	BX090412	1.48	0.001281109	1.47	0.000930605	-0.87	0.037340541	-0.88	0.025448576
	BC045163	1.45	3.62E-05	0.58	0.040103551	-1.39	3.12E-05	-2.25	3.73E-08
HOXA10	NM_018951	1.43	4.44E-06	0.86	0.000579266	-0.53	0.022134445	-1.10	2.33E-05
TLR2	NM_003264	1.42	0.000310032	1.80	1.61E-05	-2.33	7.17E-07	-1.95	2.78E-06
	THC2657493	1.38	0.000134482	1.47	4.25E-05	-2.16	4.46E-07	-2.07	3.14E-07
OASL	NM_003733	1.34	1.66E-05	0.58	0.01676382	-0.78	0.001967307	-1.54	7.42E-07
	CR625594	1.34	0.000464881	1.05	0.002484941	-0.70	0.03890031	-0.98	0.002738176
PSCA	NM_005672	1.32	0.002415497	1.59	0.000301512	-7.30	2.39E-12	-7.02	1.20E-12
	A_24_P476718	1.31	0.000870782	2.24	1.89E-06	-1.93	9.86E-06	-1.00	0.00360047
SPINK1	NM_003122	1.29	0.005034924	0.96	0.024954425	-3.40	1.10E-07	-3.73	1.34E-08
	CR612518	1.28	0.006785661	1.14	0.011308172	-0.96	0.032090928	-1.11	0.009489293
	A_32_P169353	1.20	0.045002273	1.48	0.010727271	-2.84	2.98E-05	-2.56	4.67E-05
	BC062324	1.19	0.01579769	1.22	0.010153956	-1.67	0.00082046	-1.64	0.000563989
DHX58	NM_024119	1.18	5.99E-06	0.71	0.000741093	-0.68	0.001151375	-1.15	2.09E-06
	BM989848	1.16	0.011329228	0.98	0.023865075	-0.89	0.042562201	-1.07	0.010191815
MDK	NM_001012334	1.16	6.19E-05	1.20	2.48E-05	-1.55	1.14E-06	-1.51	7.03E-07
S100A4	NM_002961	1.12	0.000446466	0.72	0.010561535	-0.77	0.007225432	-1.17	0.000100805
BTN3A2	NM_007047	1.09	1.77E-05	1.11	9.31E-06	-0.57	0.00559599	-0.55	0.004476681
ALDH1L1	NM_012190	1.09	0.006504537	1.35	0.000801896	-1.46	0.000386161	-1.20	0.001386589
SEPP1	NM_005410	1.08	0.017795719	2.85	1.26E-06	-2.78	1.49E-06	-1.00	0.01532286
	THC2671679	1.07	3.76E-05	0.73	0.001017835	-1.24	3.55E-06	-1.58	7.50E-08
HCP5	NM_006674	1.06	0.007759651	1.33	0.000898615	-1.84	3.33E-05	-1.57	9.87E-05
	AK026517	1.05	0.002902491	1.06	0.001900336	-0.80	0.015527585	-0.79	0.011579443
	THC2754005	1.02	0.000456872	1.29	2.63E-05	-1.03	0.000244525	-0.77	0.002241279
ITGA2	NM_002203	1.01	0.022552265	0.94	0.026376749	-1.06	0.012631859	-1.13	0.005479826
WNT7A	NM_004625	0.99	0.000464881	0.53	0.033957447	-1.77	4.27E-07	-2.24	8.20E-09

NEBL	NM_006393	0.99	3.91E-05	0.73	0.000558386	-0.52	0.008729268	-0.78	0.000141682
PLAT	NM_000930	0.98	0.000875467	1.35	2.25E-05	-2.14	8.14E-08	-1.77	3.78E-07
EIF2AK2	NM_002759	0.98	0.020075442	1.52	0.000512242	-1.38	0.001188256	-0.84	0.027870165
	AF086052	0.98	0.009230344	1.53	0.000138634	-1.38	0.000353702	-0.83	0.013843783
ALDH1A3	NM_000693	0.96	0.000531565	0.60	0.014677759	-1.10	8.25E-05	-1.46	1.54E-06
	BC056662	0.96	0.014386956	1.04	0.006439966	-1.04	0.006569774	-0.96	0.007518063
FMO2	NM_001460	0.96	0.037434212	2.94	1.09E-06	-3.92	2.36E-08	-1.94	5.37E-05
	A_32_P35031	0.95	0.000214111	0.62	0.005152449	-0.59	0.00820823	-0.91	0.000104052
	K03200	0.93	0.005429992	0.89	0.005220606	-1.51	3.96E-05	-1.55	1.55E-05
	AK130514	0.91	0.017250913	0.93	0.011913737	-1.39	0.000462002	-1.38	0.00028677
ZSCAN4	NM_152677	0.90	0.014937773	1.41	0.000279708	-1.54	0.000105108	-1.03	0.002697114
APOL6	NM_030641	0.89	0.018487069	1.35	0.000505697	-1.98	8.89E-06	-1.51	9.06E-05
STS	NM_000351	0.87	0.014650689	1.50	0.000108672	-2.58	1.92E-07	-1.95	2.61E-06
	THC2543120	0.86	0.009788415	1.06	0.001361535	-0.83	0.009051891	-0.63	0.035366686
PPARG	NM_138711	0.86	0.001039611	1.19	2.54E-05	-0.84	0.000819453	-0.50	0.023893587
IFITM1	NM_003641	0.85	0.002663548	0.62	0.018223872	-0.67	0.012012196	-0.90	0.000693909
	CA314451	0.84	0.016043784	0.89	0.00853883	-1.83	9.45E-06	-1.79	6.00E-06
	THC2551769	0.84	0.004358863	0.73	0.008331505	-0.69	0.012821531	-0.79	0.003004577
ENC1	NM_003633	0.83	0.002044084	1.05	0.000153929	-1.96	1.05E-07	-1.74	2.01E-07
KCNN4	NM_002250	0.82	0.006830609	1.03	0.000785576	-1.34	5.13E-05	-1.14	0.000158723
SPINK5	NM_006846	0.76	0.001825778	0.47	0.033705902	-0.70	0.002501485	-0.99	5.08E-05
MSMB	NM_002443	0.73	0.000832073	1.96	6.81E-09	-5.28	1.03E-14	-4.05	1.47E-13
PARP12	NM_022750	0.73	0.001230432	0.63	0.002667999	-1.18	4.95E-06	-1.27	8.66E-07
	THC2545454	0.71	0.02260588	0.95	0.002276893	-0.95	0.002311169	-0.71	0.012721232
LYN	NM_002350	0.71	0.003114957	1.27	7.07E-06	-1.32	3.92E-06	-0.76	0.000708175
	AK022020	0.71	0.004745127	0.59	0.012998879	-1.12	4.23E-05	-1.25	6.53E-06
LAYN	NM_178834	0.71	0.01514181	1.04	0.000500006	-1.28	5.82E-05	-0.95	0.000706483
PPP1R12B	NM_032105	0.68	0.009014906	0.68	0.00660204	-0.86	0.00096841	-0.86	0.000563989
ATP1B1	NM_001677	0.67	0.040190654	0.78	0.013887884	-0.84	0.008541899	-0.73	0.014330567
CAMK2G	NM_172171	0.66	0.009630083	0.77	0.001946373	-0.83	0.001095948	-0.71	0.002542575
	AK023696	0.64	0.030845413	0.63	0.025358095	-0.64	0.025179173	-0.64	0.017239992
TMEM132A	NM_017870	0.60	0.015740285	0.64	0.007383258	-1.72	3.16E-07	-1.68	1.76E-07
TRIM2	NM_015271	0.59	0.004377494	0.42	0.029710532	-0.50	0.009901857	-0.67	0.000579911
LRAT	NM_004744	0.51	0.043998571	0.78	0.00198053	-1.46	2.88E-06	-1.19	1.50E-05
CCNDBP1	NM_037370	0.48	0.009949949	0.67	0.00049834	-0.61	0.001068743	-0.43	0.01171219

SMAD3	U68019	0.45	0.047091148	0.52	0.017762701	-0.74	0.001205253	-0.68	0.001635126
TMEM51	NM_018022	0.45	0.028015892	0.43	0.029372403	-0.56	0.005094908	-0.58	0.002388652
	AK025166	0.39	0.028250766	0.37	0.030166685	-0.45	0.008515843	-0.47	0.004029612
NPEPPS	NM_006310	0.38	0.044356931	0.45	0.014074734	-0.79	0.000114668	-0.72	0.000155666
ALG9	NM_001077691	-0.31	0.031798838	-0.29	0.034627663	0.28	0.044702714	0.30	0.023463075
	BC002724	-0.42	0.014772148	-0.81	3.29E-05	0.93	6.18E-06	0.54	0.000991685
MPP1	NM_002436	-0.42	0.047666353	-0.78	0.000483518	1.51	2.71E-07	1.16	3.00E-06
MYO19	NM_001033580	-0.43	0.026532957	-1.00	1.21E-05	0.98	1.38E-05	0.41	0.02114696
FANCC	NM_000136	-0.51	0.011609739	-0.47	0.014584719	0.48	0.012959276	0.52	0.004986322
OVOL2	NM_021220	-0.52	0.001084197	-0.39	0.007292061	0.47	0.001844654	0.60	0.000100716
SIN3B	BC025026	-0.57	0.011569948	-0.75	0.00103124	1.01	5.47E-05	0.83	0.000227367
CDT1	NM_030928	-0.58	0.019198248	-0.90	0.000479465	1.44	2.66E-06	1.13	2.30E-05
PON3	NM_000940	-0.62	0.005105333	-0.84	0.000276722	0.93	8.48E-05	0.72	0.000647845
ADRB2	NM_000024	-0.63	0.001524882	-0.73	0.000236914	0.59	0.001792781	0.48	0.005629305
PLK1	NM_005030	-0.63	0.042808398	-0.63	0.034901844	2.40	8.38E-08	2.41	3.33E-08
	CR611847	-0.66	0.000634076	-0.51	0.003687258	0.35	0.044627807	0.50	0.002990718
	A_23_P216071	-0.70	0.00958912	-0.58	0.022202131	0.54	0.033583338	0.66	0.00706467
CTSK	NM_000396	-0.74	7.83E-05	-1.81	1.05E-09	1.64	2.75E-09	0.58	0.000331696
ASF1B	NM_018154	-0.76	0.048590758	-0.83	0.022912812	1.00	0.007299284	0.92	0.008832468
NMNAT3	NM_178177	-0.76	0.000552736	-0.87	9.23E-05	0.76	0.000345671	0.65	0.000881113
REEP2	NM_016606	-0.78	0.005759911	-0.93	0.001039901	1.69	1.66E-06	1.54	2.21E-06
ABCF2	NM_007189	-0.80	0.000944624	-0.74	0.001305162	0.60	0.00667932	0.66	0.001912744
LMCD1	NM_014583	-0.95	0.002645754	-0.76	0.010312363	0.74	0.012418953	0.93	0.001307708
NRP2	NM_201264	-0.97	0.002390052	-0.63	0.031835409	1.17	0.000297324	1.51	9.74E-06
ST7OT1	NR_002330	-0.99	0.000144723	-0.44	0.048099669	0.80	0.00068922	1.36	1.07E-06
PKMYT1	NM_182687	-0.99	0.001983455	-0.74	0.012491031	1.23	0.000180492	1.48	1.20E-05
CPT1C	NM_152359	-1.08	1.08E-05	-1.48	1.50E-07	0.94	2.56E-05	0.54	0.003391166
BEX5	NM_001012978	-1.26	0.001348714	-0.76	0.032521061	2.00	7.09E-06	2.50	2.08E-07
DHRS13	NM_144683	-1.45	0.00088615	-1.72	0.000114616	1.32	0.001366222	1.05	0.005333513
CLDN17	NM_012131	-1.47	4.17E-05	-1.79	2.57E-06	1.34	6.23E-05	1.01	0.000593411
STAC	NM_003149	-1.57	0.001093743	-1.23	0.005257075	0.88	0.043609023	1.22	0.003788264
	AK074473	-1.83	6.09E-06	-2.43	1.29E-07	1.71	7.39E-06	1.11	0.000405428
	CR602075	-2.00	0.007381353	-1.87	0.008773947	1.59	0.024722861	1.73	0.010382231
CD248	NM_020404	-2.04	2.85E-05	-1.14	0.004130168	0.77	0.049370648	1.67	7.16E-05
